# Biological and Molecular Characterization of Five *Trypanosoma cruzi* (Chagas, 1909) (Kinetoplastida, Trypanosomatidae) Isolates from the State of Hidalgo, Mexico

**DOI:** 10.3390/tropicalmed10050122

**Published:** 2025-05-01

**Authors:** Yessenia Montes-Vergara, Alberto Antonio-Campos, José Miguel Padilla-Valdez, Erick Abraham Contreras-López, Julio Cesar Noguez-García, Nancy Rivas, Ricardo Alejandre-Aguilar

**Affiliations:** 1 Laboratorio de Entomología, Escuela Nacional de Ciencias Biológicas, Instituto Politécnico Nacional, Carpio y Plan de Ayala s/n. Col. Casco de Santo Tomas, Ciudad de Mexico 11340, Mexico; ymontesv@ipn.mx (Y.M.-V.); aantonioc@ipn.mx (A.A.-C.); jmpadilla@ipn.mx (J.M.P.-V.); 2 Hospital General Regional No.25 Zaragoza, Instituto Mexicano del Seguro Social, Ciudad de Mexico 09220, Mexico; patologiatlacopan@gmail.com; 3 Área de Entomología, Laboratorio Estatal de Salud Pública de Hidalgo, Servicios de Salud Hidalgo, Pachuca de Soto 42088, Mexico; jcnog64@gmail.com

**Keywords:** *Trypanosoma cruzi*, Chagas disease, parasitemia, organotropism, genotyping, DTU

## Abstract

*Trypanosoma cruzi*, the causal agent of Chagas disease, exhibits great genetic diversity, which has been related to its biological properties. However, these are poorly known in strains from the endemic area of Hidalgo. To assess the parasite’s virulence, we evaluated parasitemia, mortality, and tropism in thirteen organs of CD1 mice during the acute phase of infection. For genotyping, we amplified the mini-exon gene from *T. cruzi* DNA using PCR. All five isolates were identified as belonging to DTU TcI. The peak of parasitemia occurred between 25 and 29 days post-infection. The Tultitlán and Olma isolates did not cause any mouse deaths, whereas Ixcatépec produced 100% mortality. Mice infected with the Barrio Hondo isolate exhibited the highest parasitemia, while those infected with Cuatecomaco had the lowest. The five isolates generated varying degrees of infection and chronic inflammation; only two isolates triggered acute pancreatitis and myocarditis. No amastigote nests were found in the hearts of mice infected with the Ixcatépec isolate. Our findings suggest that the damage caused by *T. cruzi* strains from Hidalgo may extend beyond cardiac lesions in the acute phase of Chagas disease regardless of their classification as TcI and variability in parasitemia levels.

## 1. Introduction

Chagas disease (ChD) is caused by the flagellate protozoan *Trypanosoma cruzi* (*T. cruzi*), which is transmitted by insects of the Triatominae subfamily Usinger, 1939. It is estimated that between six and seven million people worldwide are infected with this parasite [1]. In Mexico, ChD is likely underestimated as the actual number of cases is believed to be higher than reported [2]. *T. cruzi* exhibits significant genetic variability, allowing it to be classified into seven discrete typing units (DTUs): TcI-TcVI and TcBat; a DTU describes sets of stocks that are genetically more similar to each other than to any other stock and are identifiable by molecular markers [3]. While TcI is the most frequently reported DTU in Mexico, TcII, TcIII, TcIV, TcV, and TcVI have also been detected [4,5]. This genetic diversity may contribute to variations in *T. cruzi* virulence [3], which can be evaluated through factors such as the prepatent period, parasitemia, mortality, and organ tropism [6]. Different *T. cruzi* strains have shown a preference for specific tissues, leading to diverse pathological profiles [7,8]. Although tissue tropism has been questioned [9], results obtained from bioluminescence have shown differences in parasite burden even at the level of the gastrointestinal tract [10]. The heart is one of the most affected organs in ChD and has been associated with TcI in Mexico, Central, and northern South America, whereas TcII and TcV from South America have been linked to both digestive and cardiac pathologies [3]. This association between the genetic diversity of *T. cruzi* and its distribution makes DTU determination relevant.

Hidalgo is considered one of the Mexican states at potential risk for *T. cruzi* transmission, with at least one infectious triatomine species being documented [11]. To date, six different species have been reported as infectious in both domicile and peridomicile environments [12,13,14]. Additionally, the association between seroprevalence of ChD and abnormal electrocardiograms has been observed in adults and children from two localities in Hidalgo [15]. However, studies on the biological behavior and genotyping of *T. cruzi* in the state are limited. Pérez-España et al. [16] identified TcI in triatomines, while Becerril-Flores et al. [15] reported on the virulence of five strains from the El Mezquital region, where all five infected cardiac and skeletal muscle, but only two also affected the spleen and brain. However, genotyping was not performed in that study. This work aimed to biologically and molecularly characterize five *T. cruzi* isolates obtained from different localities in the Huasteca region of Hidalgo, Mexico.

## 2. Materials and Methods

### 2.1. Isolation of Trypanosoma cruzi

Five isolates of *T. cruzi* were obtained from triatomines collected in different localities from the Huasteca region of Hidalgo. Three were obtained from *T. dimidiata* (Latreille, 1811) (Tultitlán, Cuatecomaco, and Ixcatépec isolates), and two from *T. gerstaeckeri* (Stål, 1859) (Barrio Hondo and Olma isolates). Triatomine species were identified using Lent and Wygodzinsky’s keys [17], and the updated key of the genus *Triatoma* for species reported in Mexico [18]. Natural infection with *T. cruzi* was determined by examining fecal samples from the triatomines collected through spontaneous defecation following their feeding on New Zealand rabbits. Positive fecal samples from each triatomine were used to inoculate two female CD1 mice intraperitoneally (n = 10 animals in total). Fourteen days post-inoculation, blood samples were taken from the caudal vein and examined under a DM500 optical microscope (Leica, Heerbrugg, Switzerland) at 40× magnification to assess the presence of metacyclic trypomastigotes. Once the infection was confirmed, a group of 30 first-instar *Triatoma pallidipennis* nymphs were fed on the infected mice to maintain the strain.

### 2.2. Parasitemia Curves

Groups of six 9–12 week-old female CD1 mice weighing 25 ± 2 g were inoculated intraperitoneally with 5 × 10^3^ metacyclic trypomastigotes from each of the five *T. cruzi* isolates (n = 30 animals in total). The number of animals used per group was the minimum necessary recommended to assess parasitemia [19]. The blood parasite concentration was determined using the modified Pizzi–Brener technique [20]. Briefly, 6 μL blood samples were collected from the caudal vein of each mouse, and 20 fields per coverslip (18 × 18 mm) were randomly examined microscopically at 40× magnification. Parasite counts were performed for all isolates on alternate days, from day 8 to day 60 post-inoculation, and mortality was recorded over time. A control group (n = 3) was inoculated with a 0.9% saline solution following the same protocol.

Mice were maintained in polycarbonate cages (three per cage) with pine shavings as beds under 12 h light/dark cycles, and a temperature of 22 ± 2 °C, with free access to food and water throughout the study. All animal procedures were conducted under the guidelines of the Mexican Official Norm (NOM-062-ZOO-1999) and approved by the Instituto Nacional de Cardiología ethics committee as part of project number 16-964.

### 2.3. Histopathological Analysis

For this experiment, a total of 18 animals were used; groups of three CD1 mice were infected with each of the five *T. cruzi* isolates, and a control group was inoculated following the same protocol used for the parasitemia curves. The animals were euthanized through cervical dislocation at the peak of parasitemia. The brain, heart, right lung, esophagus, liver, spleen, right kidney, pancreas, urinary bladder, skeletal muscle (femoral quadriceps), stomach, small intestine, and colon were harvested and fixed in 10% formaldehyde. The histotechnological procedure was performed according to the methods described by AFIP [21]. Tissue samples were placed in embedding cassettes and processed using a TP1020 automatic tissue processor (Leica, Nussloch, Germany) for dehydration and pre-impregnation. Subsequently, samples were embedded in paraffin, and three sections (2–3 μm each) were obtained from each block and stained with hematoxylin–eosin (H&E). In each tissue sample, we examined the presence of amastigote nests and inflammatory lesions under a CX23 optical microscope (Olympus, Tokyo, Japan) at 40× magnification; both were semi-quantitatively classified as follows: scarce (+), moderate (++), and abundant (+++).

### 2.4. Trypanosoma cruzi DNA Extraction

For genotyping, Ninoa (MHOM/MX/1986/Ninoa) [22] and Y (MHOM/BR/1950/Y) [23] strains of *T. cruzi* were used as positive controls. These strains were maintained in brain–heart infusion (BHI) medium supplemented with 1% decomplemented fetal bovine serum. Control parasite pellets were washed with 1 mL of 1X PBS and then centrifuged at 6000 rpm for 5 min. The supernatant was discarded, and the sediment was stored at −20 °C until further use.

DNA extraction from *T. cruzi* was performed using a modified version of the salting-out method described by Riera et al. [24] to accommodate triatomine urine samples. For digestion, each urine sample (1.5 mL) and the control samples were thawed at room temperature. To each sample, 400 μL of lysis buffer (50 mM EDTA pH 8, 50 mM Tris-HCl pH 8, 50 mM NaCl, and 1% SDS) and 10 μL of 20 mg/mL proteinase K were added and thoroughly mixed. Samples were incubated at 65 ± 2 °C for 4 h, with intermittent mixing each hour. Following incubation, samples were centrifuged at 6000 rpm for 5 min, and the supernatant was transferred to new tubes. For protein and cell debris precipitation, 3/10 of 3 M sodium acetate (pH 5.2) was added to the supernatant, mixed, and centrifuged at 11,000 rpm for 5 min. The supernatant was transferred to new tubes, followed by a second wash to recover the supernatant. DNA precipitation was performed by adding two volumes of isopropanol, mixing via inversion, and centrifuging at 11,000 rpm for 6 min. The supernatant was discarded. DNA was further washed with 800 μL of 70% ethanol, followed by centrifugation at 11,000 rpm for 10 min. After discarding the ethanol, the DNA pellet was air-dried, resuspended in 30 μL of sterile ultrapure water, and stored at −20 °C until use.

### 2.5. Genotyping of Trypanosoma cruzi Isolates

The intergenic region of the mini-exon gene (SL-IR) of *T. cruzi* was amplified using a primer pool designed by Souto et al. [25]. The primers included the following: TcI forward primer, 5′-GTGTCCGCCACCTCCTTCGGGCC-3′ (TcI); a common forward primer for TcII-TcVI, 5′-CCTGCAGG-CACACGTGTGTGTG-3′ (TcII); and a common reverse primer for TcI-TcVI 5′-CCCCCCTCCCAGGCCACACTG-3′ (Tc). The 20 μL PCR reaction mix consisted of 2 μL of DNA template, 2 μL of 10X PCR buffer, 1.25 μL of 50 mM MgCl_2_, 0.25 μL of Taq DNA polymerase (500 U/μL, Invitrogen, Carlsbad, CA, USA), 2 μL of 2 mM dNTPs (Vivantis, Shah Alam, Malaysia), 0.5 μL each of 10 mM primer (T4 OLIGO, Guanajuato, Mexico), and 11 μL of ultrapure water. The PCR protocol was carried out as described by Padilla-Valdez et al. [26]. The amplified products were separated via electrophoresis on a 2% agarose gel at 100 V, stained with ethidium bromide, and visualized under a UV transilluminator (ChemiDoc Imaging System, BIO-RAD, Hercules, CA, USA). The Ninoa and Y strains, corresponding to TcI and TcII, respectively, were used as positive controls. Sterile ultrapure deionized water was used as a negative control, and a 1 Kb Plus DNA ladder (Invitrogen, Carlsbad, CA, USA) was used as a molecular size marker.

## 3. Results

### 3.1. Prepatent Period, Parasitemia, and Mortality

**Figure 1:**
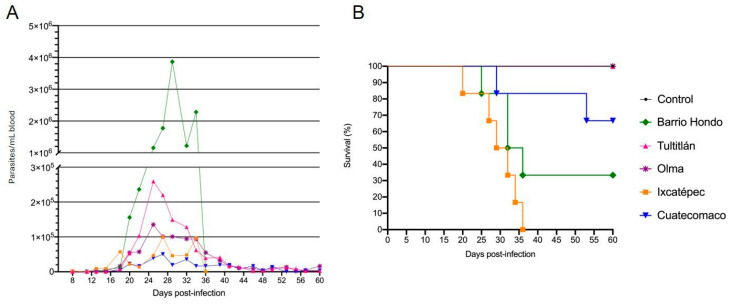
Biological behavior of *T. cruzi* isolates from the state of Hidalgo in mice. (**A**) Parasitemia curves and (**B**) survival curves. CD1 mice were inoculated with 5 × 10^3^ parasites/mL (n = 6).

Mice inoculated with the Tultitlán and Olma isolates exhibited a parasitemia peak 25 days post-infection, with the Tutitlán isolate inducing a higher parasitic load (259 × 10^3^ trypomastigotes/mL of blood). The prepatent period for Tultitlán was 18 days, compared with 13 days for the Olma isolate (Figure 1A). There was no mortality in either group during the infection period (Figure 1B). For the Ixcatépec and Cuatecomaco isolates, parasitemia began on days 8 and 18, respectively, with both groups reaching peak parasitemia on day 27 post-infection. The Cuatecomaco parasitemia curve remained low but consistent throughout the experiment, fluctuating between 35 × 10^3^ and 50 × 10^3^ trypomastigotes/mL of blood during the days with higher parasitemia (Figure 1A). Despite relatively low parasitemia, two mice died on days 29 and 53, resulting in a 67% survival rate (Figure 1B). Mice infected with the Ixcatépec isolate developed posterior limp weakness before dying within 15 days (Figure 1B). Although the Barrio Hondo isolate produced the highest parasitemia (3.9 × 10^6^ trypomastigotes/mL of blood), only four mice died, corresponding to a 33% survival rate. Parasites were first detected on day 15 post-infection, with peak parasitemia occurring on day 29. No parasites were observed in surviving animals by day 36 post-infection (Figure 1A). During the infection period, urinary and fecal incontinence was noted in mice infected with the Tultitlán and Barrio Hondo isolates.

### 3.2. Organotropism

**Figure 2:**
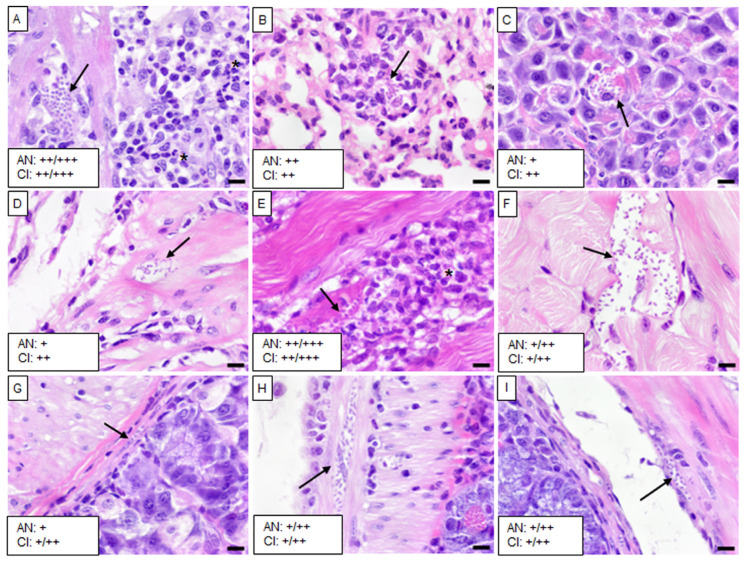
Histological sections of tissues from mice infected with the Barrio Hondo isolate. (**A**) Heart, (**B**) right lung, (**C**) pancreas, (**D**) urinary bladder, (**E**) skeletal muscle, (**F**) esophagus, (**G**) stomach, (**H**) small intestine, and (**I**) colon. Black arrows indicate *T. cruzi* amastigote nests, and asterisks denote acute inflammatory infiltrates. Scale bar: 20 µm. H&E staining, 40× magnification. The small boxes indicate the semiquantitative assessment of amastigote nests (AN) and chronic inflammation (CI): scarce (+); moderate (++); scarce/moderate (+/++); moderate/abundant (++/+++).

**Figure 3:**
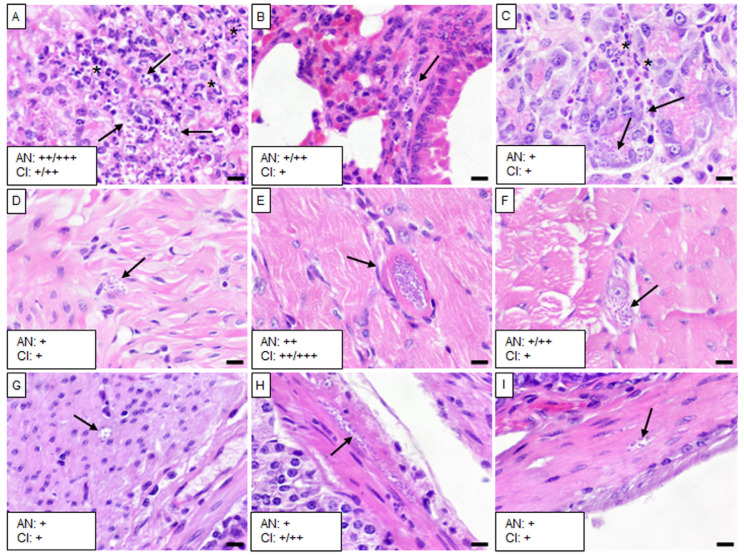
Histological sections of tissues from mice infected with the Olma isolate. (**A**) Heart, (**B**) right lung, (**C**) pancreas, (**D**) urinary bladder, (**E**) skeletal muscle, (**F**) esophagus, (**G**) stomach, (**H**) small intestine, and (**I**) colon. Black arrows indicate *T. cruzi* amastigote nests, and asterisks denote acute inflammatory infiltrates. Scale bar: 20 µm. H&E staining, 40× magnification. The small boxes indicate the semiquantitative assessment of amastigote nests (AN) and chronic inflammation (CI): scarce (+); moderate (++); scarce/moderate (+/++); moderate/abundant (++/+++).

The five isolates caused varying levels of infection with *T. cruzi* and chronic inflammation in different tissues, except in the brain, liver, spleen, and kidney. Mice infected with the Barrio Hondo and Olma isolates showed a considerable number of amastigote nests in the heart, particularly in the myocardium of auricles and ventricles, along with chronic and acute inflammation and disorganized fibers (Figure 2A and Figure 3A).

In these mice, low to moderate numbers of amastigote nests were also found in the lungs, specifically in the pulmonary septum (Figure 2B) and the bronchial and vascular smooth muscle (Figure 3B). The Barrio Hondo and Olma isolates induced moderate and mild chronic inflammation, respectively. Additionally, we found evidence of acute pancreatitis with sparse amastigote nests in acinar cells (Figure 2C and Figure 3C). No amastigote nests were observed in the heart and lungs of mice infected with the Ixcatépec isolate.

**Figure 4:**
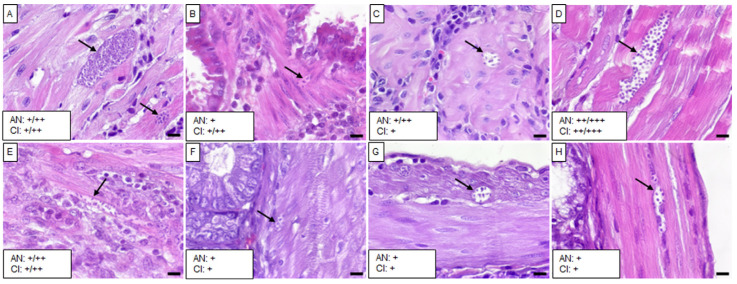
Histological sections of tissues from mice infected with the Tultitlán isolate. (**A**) Heart, (**B**) right lung, (**C**) urinary bladder, (**D**) skeletal muscle, (**E**) esophagus, (**F**) stomach, (**G**) small intestine, and (**H**) colon. Black arrows indicate *T. cruzi* amastigote nests in the tissues. Scale bar: 20 µm. H&E staining, 40× magnification. The small boxes indicate the semiquantitative assessment of amastigote nests (AN) and chronic inflammation (CI): scarce (+); scarce/moderate (+/++); moderate/abundant (++/+++).

**Figure 5:**
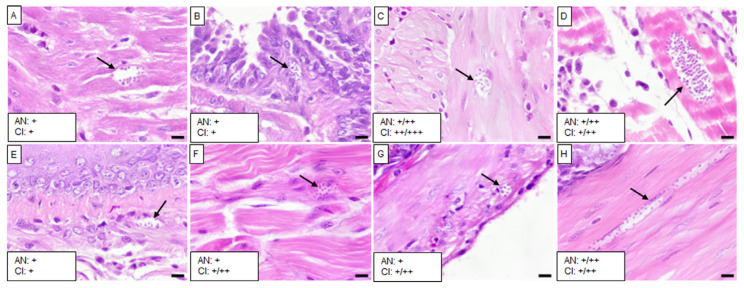
Histological sections of tissues from mice infected with the Cuatecomaco isolate. (**A**) Heart, (**B**) right lung, (**C**) urinary bladder, (**D**) skeletal muscle, (**E**) esophagus, (**F**) stomach, (**G**) small intestine, and (**H**) colon. Black arrows indicate *T. cruzi* amastigote nests in the tissues. Scale bar: 20 µm. H&E staining, 40× magnification. The small boxes indicate the semiquantitative assessment of amastigote nests (AN) and chronic inflammation (CI): scarce (+); scarce/moderate (+/++); moderate/abundant (++/+++).

**Figure 6:**
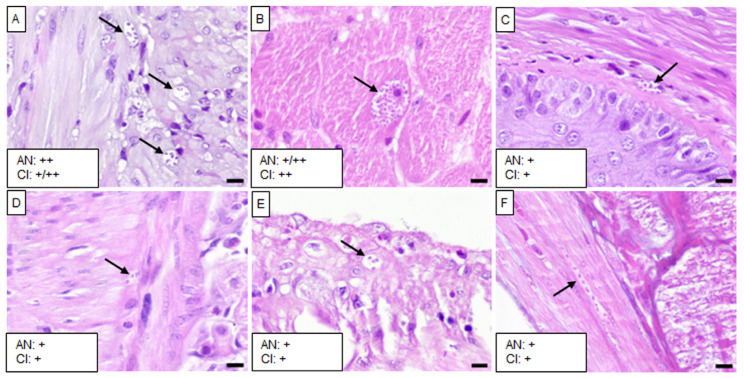
Histological sections of tissues from mice infected with the Ixcatépec isolate. (**A**) Urinary bladder, (**B**) skeletal muscle, (**C**) esophagus, (**D**) stomach, (**E**) small intestine, and (**F**) colon. Black arrows indicate *T. cruzi* amastigote nests in the tissues. Scale bar: 20 µm. H&E staining, 40× magnification. The small boxes indicate the semiquantitative assessment of amastigote nests (AN) and chronic inflammation (CI): scarce (+); moderate (++); scarce/moderate (+/++).

Mice infected with the Tultitlán, Cuatecomaco, and Ixcatépec isolates showed sparse to moderate amastigote nests in the detrusor muscle of the urinary bladder, with nest sizes ranging from 19 to 50 µm (Figure 4C, Figure 5C and Figure 6A). Only the Cuatecomaco isolate induced moderate to intense inflammation in the detrusor muscle. Skeletal muscle was primarily infected with the Barrio Hondo and Tultitlán isolates, where moderate to abundant amastigote nests were observed, along with chronic inflammation, which was also noted with the Olma isolate. Acute myositis developed only in mice infected with the Barrio Hondo isolate (Figure 2E). In general, the amastigote nests found in skeletal muscle were considerable in size (65–164 µm, with some exceeding 200 µm), containing many parasites and even compacting cytoplasmic content at the periphery (Figure 3E).

In the case of the upper gastrointestinal tract (GIT), mice infected with the Barrio Hondo, Olma, and Tultitlán isolates presented scarce to moderate amastigote nests in the muscularis mucosae and the tunica muscularis of the esophagus. Only the Olma isolate caused infection in ganglion cells (Figure 3F), while the Tultitlán and Barrio Hondo isolates induced mild to moderate chronic inflammation. All *T. cruzi* isolates produced scarce amastigote nests of a small size (15–43 µm) in the stomach. Infection was more pronounced in the colon of animals infected with the Barrio Hondo and Cuatecomaco isolates (Figure 2I and Figure 5H, respectively), and in the small intestine of mice infected with the Barrio Hondo isolate (Figure 2H) where elongated amastigote nests were found along the muscularis mucosae and tunica muscularis, accompanied by mild to moderate inflammation.

### 3.3. Molecular Characterization of T. cruzi Genotypes

**Figure 7:**
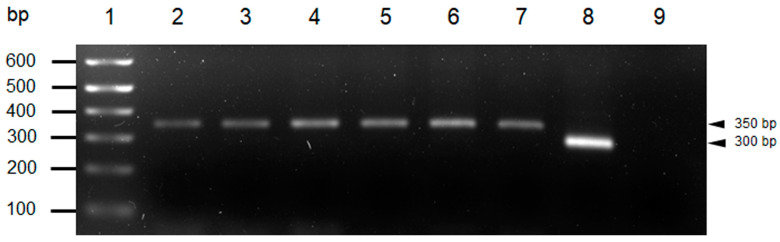
PCR products of T. cruzi mini-exon from the five isolates. Each lane represents the following: (1) molecular marker, (2) Tultitlán, (3) Ixcatépec, (4) Cuatecomaco, (5) Barrio Hondo, (6) Olma, (7) positive control Ninoa (TcI, 350 bp), (8) positive control Y (TcII-TcVI, 300 bp), and (9) negative control.

PCR amplification of the *T. cruzi* mini-exon gene produced a 350 bp band on the electrophoresis gel corresponding to the TcI genotype, which was consistent with the Ninoa strain used as a positive control. In contrast, the Y strain, representing the TcII-TcVI genotypes, produced a 300 bp band (Figure 7).

## 4. Discussion

Hidalgo, a Mexican state, is considered an endemic area for Chagas disease (ChD). However, limited information exists on the overall impact of circulating *Trypanosoma cruzi* strains on the host despite their wide genetic diversity, which has been linked to varying biological properties [3]. In this study, five *T. cruzi* isolates from different locations in the Huasteca region of Hidalgo were characterized. Parasitemia analyses revealed significant differences between mice infected with the Barrio Hondo and Olma isolates from *Triatoma gerstaeckeri* and those infected with the Tultitlán, Ixcatépec, and Cuatecomato isolates from *Triatoma dimidiata*. The parasitemia peaks of the Barrio Hondo and Olma isolates were 29 times higher than those of the latter isolates. Mice infected with Mexican isolates obtained from *Triatoma pallidipennis* have also exhibited differences in parasitemia; while some show more than 4 × 10^6^, others do not reach one million [7]. Something similar has been seen with strains isolated from human patients [27]. Previous reports indicate that parasitemia peaks occur between 21 and 29 days post-infection [7,15], aligning with the findings of this study.

The Barrio Hondo isolate was one of the most pathogenic, causing high parasitemia and resulting in the death of more than 50% of the infected mice. In contrast, although the Ixcatépec isolate induced lower parasitemia levels, it surprisingly caused 100% mortality in infected mice. This kind of response has also been seen with Brazilian strains isolated from human patients [28]. *T. cruzi* is known to express a wide array of virulence factors on its surface at different stages of its life cycle [29]. However, due to intragenomic variability in the parasite’s disruptive regions, these virulence factors can change, influencing not only the discrete typing units (DTUs) but also variations in intra-DTUs [3]. This genetic variability in multicopy genes may explain heterogeneity in the behavior of the isolates, particularly in terms of parasitemia, mortality, and organotropism.

Previous studies on mice infected with Mexican *T. cruzi* strains belonging to the TcI genotype from different triatomine species have shown that during the acute phase of ChD, the myocardium of the auricles and ventricles is the most affected heart tissue, exhibiting varying degrees of mononuclear cell infiltration [8,30]. These infiltrates consist primarily of immune cells involved in the host response against *T. cruzi* [31,32]. Notably, the Barrio Hondo and Olma isolates also promoted the recruitment of polymorphonuclear cells in the heart. Molina and Kierszenbaum [33] demonstrated that this kind of leucocyte causes damage in cardiac cells in the presence of *T. cruzi* amastigotes through the secretion of myeloperoxidase.

In contrast, no leucocyte infiltrates were detected in the cardiac tissue of mice infected with the Ixcatépec isolate, likely due to the absence of amastigote nests. Previous research also identified *T. cruzi* surface glycoprotein-85 (gp-85) as being involved in cell invasion [34]. A mutagenized version of FLY, a conserved peptide in the gp-85/trans-sialidase family, showed reduced binding to cytokeratin-8 (CK-8) expressed in heart muscle cells, which may explain the low parasitic load observed in heart tissue.

Although *T. cruzi* can infect a wide range of nucleated cells, many strains show a marked affinity for skeletal, cardiac, and smooth muscle [7,32] as seen with the isolates characterized in this study. It could be possible that morphofunctional characteristics shared among the three types of muscle play a significant role during infection. Studies have shown that in epithelial, endothelial, and fibroblast cells, the presence of trypomastigotes induces activation of nuclear factor-kB (NF-kB), a transcription factor involved in immune responses. In contrast, this activation is absent in muscle cells, which may contribute to increased parasitemia in these tissues [35]. In addition to the immune response, the host metabolic adaptation role has also been analyzed as a key to determining *T. cruzi* tissue tropism since some metabolites can benefit its proliferation and persistence in certain tissues, like upregulation of glucose uptake in myoblasts [36]. Part of the loss of mobility observed in the hind limbs of mice infected with the Ixcatépec isolate could be attributed to moderate local inflammation in the femoral quadriceps. However, peripheral nerves, such as the sciatic and sural nerves, are also affected during ChD, reducing muscle responsiveness in both mice and humans [37].

Other Mexican TcI strains have also demonstrated varying degrees of tissue invasion in the heart, skeletal muscle, gastroesophageal junction [7], brain [8], and lungs [38] during the acute phase of ChD. However, the urinary bladder is one of the least studied organs in this context. In the acute phase of ChD, amastigote nests have been observed not only in the detrusor muscle but also in other layers of the urinary bladder in mice infected with the Y strain (TcII) [39], while the CL-Brener strain (TcVI) has shown urothelial cell invasion [40]. Additionally, findings from Boczko et al. [41] suggest that the bladders of CD1 mice are overactive in the chronic phase of ChD, potentially due to inflammatory processes initiated during the acute phase. Based on both our observations and histopathological evaluations, it is possible that the Tultitlán, Ixcatépec, Cuatecomaco, and Barrio Hondo isolates could trigger a megabladder in the chronic phase of ChD as previously described [39].

In general, the esophagus was one of the segments of the gastrointestinal tract (GIT) that was most heavily infected by the isolates, followed by the colon, small intestine, and stomach. In mice infected with the CL-Brener strain, it has also been seen that the esophagus is one of the segments with a higher parasite burden, in addition to an increase in molecules like acylcarnitine and phosphatidylcholine during the acute phase of ChD that persists until the chronic phase [10], which could be related to a metabolic adaptation in this specific site of the GIT [36]. Recently, Flores-Villegas et al. [8] observed amastigote nests in the esophagus of mice infected with TcI strains from Oaxaca. Similarly, Espinoza et al. [32] found parasites in all three segments of the small intestine, and the colon of mice also infected with TcI strains. A key observation is that the amastigote nests were located near Auerbach’s plexus. This is significant as neurodegeneration of the GIT has been linked to direct parasitism of the myenteric and submucosal plexuses during the acute phase of ChD [42]. Additionally, the isolates produced varying degrees of mononuclear cell infiltration in the GIT, particularly with the Cuatecomaco and Barrio Hondo isolates. It is known that the invasion of immune cells and the production of pro-inflammatory cytokines have been implicated in neurodegeneration within the GIT plexuses [42]. Given that denervation is considered the primary pathogenic mechanism in the chronic form of ChD affecting the GIT, and with fecal incontinence observed in mice infected with the Tultitlán and Barrio Hondo isolates, it is suggested that the isolates characterized in this study have significant potential to induce gastrointestinal disorders.

The Barrio Hondo and Olma parasites were observed to infect acinar cells, leading to acute pancreatitis, a pathology also reported in infections with the CL-Brener strain (TcVI) [40] and Y strain (TcII) [43]. During acute pancreatitis, trypsinogen activation occurs within acinar cells themselves, alongside activation of NF-kB, which triggers the release of inflammatory mediators and the recruitment of immune cells [44]. It has been shown that *T. cruzi* can activate NF-kB through Toll-like receptor 2 (TLR2) via GPI–anchored mucin-like glycoproteins on the parasite’s surface [45], suggesting that acute pancreatitis could begin before the parasite enters the cell.

In this study, we did not observe amastigote nests in the brain, liver, spleen, and kidney with any of the five isolates. Strains obtained in the El Mezquital region from the state of Hidalgo have also shown differences in tropism; no strain infected the liver, and not all strains infected the spleen and brain [15]. Amastigote nests have been seen in the cerebellar gray matter and astrocytes of animals infected with the Y strain [46]; however, in mice infected with the Colombian strain (TcI), amastigote forms were not detected in the central nervous system except in one mouse [47]. These differences could be due to the specific behavior of each strain, combined with the significant role of the immune response. Additional studies are necessary to elucidate the molecular mechanisms involved in infection with the characterized isolates.

Genotyping revealed that all five isolates belong to DTU TcI, which is consistent with findings from other studies on Mexican strains where *T. cruzi* has been isolated from both *T. dimidiata* [48,49] and various other species of triatomines (except for *T. gerstaeckeri*), as well as from human and animal reservoirs [7,50,51]. In the state of Hidalgo, TcI has been reported in ten municipalities [16], including San Felipe Orizatlán, Huejutla de Reyes, and Huautla, where three of the isolates in this study were obtained. While all five isolates were genotypically TcI, they exhibited distinct biological behaviors. Due to genetic variability in the intergenic region of the mini-exon, TcI has been subdivided into five genotypes (TcIa, TcIb, TcIc, TcId, and TcIe), each associated with different transmission cycles of ChD [52,53]. In Colombia, Cruz et al. [54] observed biological differences between domestic TcI (TcIa) and sylvatic TcI (TcId) strains in CD1 mice. In addition, López-Vivas et al. [55] identified TcIa in parasite samples from *T. pallidipennis* in the state of Mexico, although virulence was not evaluated. It is plausible that the isolates characterized in this study belong to a specific TcI subdivision, but further experiments are necessary to confirm this hypothesis.

## 5. Conclusions

The results indicate that the damage caused by *T. cruzi* strains from Hidalgo extends beyond cardiac lesions in the acute phase of ChD, regardless of their classification as TcI and variations in parasitemia levels. In patients, this would imply that the search for damaged tissue should not be limited to the heart alone. Due to the cascade of events initiated during the acute phase, it is important to monitor other organs as they may also be susceptible to developing disorders in the chronic phase of ChD. These findings could contribute to establishing a long-term clinical prognosis for the disease in the endemic region of Hidalgo. Furthermore, this study highlights that strains with low parasitemia are not necessarily less lethal, emphasizing the need for continued research on the molecular mechanisms underlying *T. cruzi* virulence and further molecular characterization to enhance our knowledge of TcI.

## Data Availability

The original contributions presented in the study are included in the article; further inquiries can be directed to the corresponding author.
